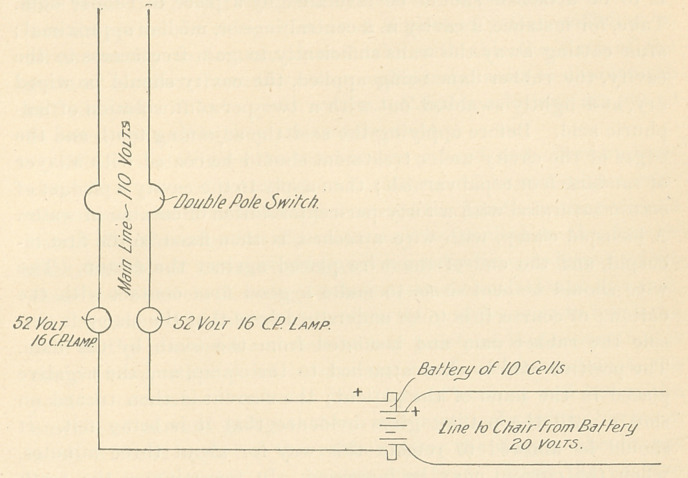# Suggestions on the Use of Electrical Osmosis in Dental Practice

**Published:** 1896-04

**Authors:** Peter Brown

**Affiliations:** 14 Phillip’s Square; Montreal, Canada


### International Dental Journal.

Vol. XVII. April, 1896. No. 4.

### Original Communications.[1]

### SUGGESTIONS ON THE USE OF ELECTRICAL OSMOSIS IN DENTAL PRACTICE.

BY PETER BROWN, L.D.S., MONTREAL, CANADA.

The efficiency of this treatment in obtunding sensitive dentine has been so abundantly proved by other writers that any further argument in this article would be superfluous.

One of the principal things to be considered now is its method of application and the source of energy to be used. We have many sources of electricity, and in this article an effort will be made to suggest the best means of obtaining satisfactory results in cataphoric medication of sensitive dentine.

One source of power which is very much in use by dentists to-day is the ordinary one-hundred-and-ten-volt lightning circuit; this is a very satisfactory means of running a dental engine or a mallet, but it is far from satisfactory for the treatment under consideration. It is open to quite a few objections. Prominent among them is the unevenness of the voltage; for the delicate nature of the application in treating sensitive dentine a perfectly even pressure of current is absolutely necessary. This evenness is impossible to obtain on the ordinary light or power circuit, owing to the varying de-

[1] The editor and publishers are not responsible for the views of authors of papers published in this department, nor for any claim to novelty, or otherwise, that may be made by them. No papers will be received for this department that have appeared in any other journal published in the country.

mands made upon the generators from time to time during the day. A difference in potential of five per cent. may not be of much importance to the central station supplying the circuit, but it is of great importance to the patient who is being treated by it. From a large number of experiments made by the writer it has been proved that a difference in pressure of one-fifth of a volt is immediately felt by the patient, and very often it is painfully felt; this shows the necessity of a steady current. Another objection is that there is great danger from wires of a higher pressure falling across, or otherwise coming in contact with, the line supplying the dentist; this has happened more than once, and very often with unpleasant results. The fact of having a resistance in the circuit will not afford protection from accidents of this kind, as the sudden increase of pressure will overcome the resistance in use at the time, and force more current through than is required before additional resistance can be added. The danger of high-pressure circuits may be avoided by reducing the voltage with what is known as a rotary transformer. The type known as the motor-dynamo should not be used, as there is a danger here from leakage from the high-pressure side of the machine to the low-pressure, which is the one to be used in treatment; a one-hundred-and-ten-volt circuit may be reduced by connecting a one-hundred-and-ten-volt motor by means of a belt to a dynamo wound to give a current of twenty volts. The current from this could then be led to the chair, and used without the slightest fear of shock. This plan is, however, open to the objection of varying pressures, as when the voltage of the main line would drop there would be a drop in speed of the motor and a corresponding drop in the voltage of the dynamo. In the writer's experience, the most satisfactory and most reliable source of power for this work is the storage-battery. Where one can obtain the one-hundred-and-ten-volt circuit there is very little or no trouble in operating it; when it is received from the manufacturers it is charged, ready for use, and it may be connected with the one-hundred-and-ten-volt circuit with a sixteen candle-power lamp in series with it for resistance; as in sketch, a double pole-switch should be put in between the line and the lamp, and always opened when the battery is in use. Here we have a safe and reliable supply of energy always at our command, and always at an even pressure. The battery is kept charged by the lighting circuit, and requires but very little attention. A battery of ten cells should be employed, so as to have a potential of twenty volts; this is all the voltage necessary for this work, and is not very often required. Ten volts will be found quite sufficient for the majority of cases, but is advisable to have an extra pressure in case of patients who have a very high resistance. Where the direct current is not available the battery can be charged at the nearest lighting station, and one charge should last a dentist for cataphoric treatment at least six months; where it is not desirable to go to the expense of buying a storage battery the ordinary Le Clanche cell of the type used for operating the telephone would answer the purpose very well, or the bichromate of soda cell makes a good battery, but they are more troublesome than the storage cell.

The next thing to consider is the means of reducing the current so that it can be tolerated by the tooth under treatment. This is accomplished by placing a rheostat in series with the battery and patient. The most compact and reliable form of resistance, in the writer's estimation, is one called the "Williams dry current-controller." This will give a resistance of one hundred thousand ohms on a ten-volt circuit, thus giving about the one tenth of a milliampère on the first contact. A milliampère meter is not necessary, but is very useful for purposes of record, and for assuring one that the current is on when the contacts are made. A voltmeter is also very useful for recording the pressure, but both of these instruments can be dispensed with. Where a galvanometer and voltmeter are used instruments recording in tenths or twentieths should be employed. It is of great importance that the contact with the tooth should be firm, and not removed until the operation is completed, as any break in the circuit when the current is on is accompanied by quite a severe shock; before removing the contact from the tooth the current should be reduced by the rheostat to its zero point. A very good method of obtaining good contact with the tooth is to solder or otherwise attach to a rubber-dam clamp a piece of brass wire. To the end of this wire a piece of platinum should be soldered, as no metal but platinum should be used in contact with the tooth. In applying the clamp the tooth to which it is to be attached should be insulated by a piece of rubber dam. Take, for instance, a cavity in a central incisor, median approximal; after cutting away the walls sufficiently to gain free access to the cavity, the rubber dam being applied, the cavity should be wiped dry, and lightly swabbed out with a two-per-cent. solution of sulphuric acid. Before applying the acid the adjoining tooth and the edges of the cavity under treatment should be coated with a layer of sandarach or copal varnish; then apply to the cavity a pledget of cotton saturated with a forty-per-cent. solution of cocaine in water. A bicuspid clamp, with wire attached, is then fixed to the first bicuspid, and the end of the wire placed against the cotton. The wire should be bent so as to make a good firm contact with the cotton; of course, it is to be understood here that the clamp is outside the rubber dam and insulated from the tooth by the dam. The positive wire is then attached to the clamp, and the negative placed in the hand of the patient; the current is then turned on slowly until the patient gives evidence that it is being felt. It should be allowed to remain this way for about three minutes, when the current may be increased. In ten minutes the tooth should be insusceptible to pain, although the writer has had cases where forty minutes were necessary to obtund the dentine, but these cases are exceptional. In operating, where there are other fillings in the same tooth, and where these fillings are liable to be touched in preparing the cavity, a coating of varnish should be put on so as to insulate them.

Where it is desirable to remove the pulp, the following method may be used: Prepare the tooth and cavity as before mentioned, but cover the exposed pulp with sticking-plaster or other suitable substance; then place the cotton containing the cocaine in the cavity and apply the current as before. In ten minutes the current may be turned off, and the cotton removed, and the covering taken from the pulp.

Another application may be then placed in the cavity in direct contact with the pulp, and the current again applied. In ten or fifteen minutes the pulp can be removed without any pain, and the root immediately filled. In all these operations the cotton containing the solution should be moistened every four or five minutes, as when the water evaporates the current does not pass through, and time is wasted. A drop of water may be added with a small syringe or applied with a piece of cotton without removing the wire from the cavity.

Attention is directed to the following: Always make a fresh mixture of cocaine; have one grain of cocaine weighed out and divided into six parts. In using it moisten a piece of cotton sufficient to fill the cavity to be treated, and take with this the sixth of the grain of cocaine; have the cotton sufficiently moist to dissolve the salt and you will have good results. Cocaine solutions are not to be relied upon after they are two days old.

Where the skin on the hand is dry or hard and offers high resistance, a piece of cottonoid or lintine should be wet with a solution of common salt and wrapped around the handle in the patient's hand. Or, the negative electrode may be applied to the face or neck, but this is not desirable, as it leaves quite a red spot where the contact was made, owing to the increased circulation at this part.

Care should be taken not to allow the positive wire to touch the skin or the mucous membrane at any point, as, if a connection is made at any other point besides the tooth under treatment, the positive current will enter at this place of contact and lessen the amount of current going through the tooth. In using this treatment without applying the rubber dam, the fingers should not come in contact with the mucous membrane unless the positive electrode has a well-insulated handle.

The following cases may be of interest:

Mr. B., aged twenty, highly nervous, presented a first left superior bicuspid anterior approximal cavity; history of toothache for two days; on examination, pulp was found exposed; the dam was applied and cavity wiped dry. At this time the tooth was giving severe pain. A pledget of cotton saturated with the cocaine solution was applied, and the current turned on. In less than one minute the pain had ceased; the current was allowed to pass through for fifteen minutes. At the end of this time the application was removed and the pulp was found completely anæsthetized; it was removed with broach and drill, and the cavity immediately filled.

Mr. H., aged eighteen, approximal cavity in central incisor exceedingly sensitive; could not bear the slightest touch of excavator or bur. At this time the writer was using a water rheostat and the current was derived from a ten-volt dynamo; by reducing the speed a current of five volts was attainable. This was the voltage of the current first used on this case. With the rheostat set at its highest resistance the patient could not stand the current, giving marked evidence of pain on the first contact. Later, a dry current controller was procured, and the current taken from a storage battery of ten cells. This battery was provided with a cell-selector or switch-board by which the voltage could be varied from two to twenty volts. The same case was then treated; the current had to be first reduced to the pressure from one cell or two volts; then again reduced by the rheostat. This current was easily tolerated by the patient, and was increased, after ten minutes' application, to ten volts. It was found necessary to treat this tooth for thirty minutes before the dentine was anæsthized. Six cavities were filled for this patient, and each one of them required the same treatment.

Miss G., cavity in second superior molar anterior approximal; pulp exposed; history of toothache for three or four days. The dam was applied, and the cocaine applied as before, only in this case the exposure was protected by a coating of copal varnish. After about eight minutes' application of the current the cotton was removed, and the application placed in direct contact with the pulp. The current was again applied, and allowed to remain on for twenty minutes. At the end of this time the pulp was found completely insensible to pain, and was removed without any pain or trouble.

Mrs. L., two approximate cavities in central incisor and right lateral approximal to each other; after applying the dam the walls of cavities were slightly cut away so as to allow free access to them, and the space between them filled with cotton saturated with the cocaine solution. A clamp was attached to the first bicuspid, and the wire, which was attached to this clamp, pressed into the cotton. The current was then turned for ten minutes and both cavities were anæsthetized at the same time. The cavities were both filled at this sitting and were found to be insensible to the pain which very often accompanies the operation of finishing gold fillings with strips or disks. Advantage should be taken of this, and cavities always filled at the same sitting as when prepared, especially for gold fillings.

Miss M., cavities in both superior left bicuspids; after the dam was applied to these teeth only, a clamp was put on the first molar. To this clamp was soldered a wire having two branches from its end; these were tipped with platinum. After cutting away the edges of the cavities and placing the cocaine in them, the two ends of the wire were arranged so as to have a tip in each cavity. By this means both teeth were treated at once, and much time saved.

14 PHILLIP'S SQUARE.

## Figures and Tables

**Figure f1:**